# Attitudes to drug trials among relatives of unconscious intensive care patients

**DOI:** 10.1186/1471-2253-10-6

**Published:** 2010-05-26

**Authors:** Anders Perner, Michael Ibsen, Jan Bonde

**Affiliations:** 1 Department of Intensive Care, Copenhagen University Hospital, Rigshospitalet, Denmark

## Abstract

**Background:**

In many countries relatives are asked to consent on behalf of ICU patients prior to inclusion in clinical trials. However, the attitudes to drug trials among relatives of unconscious ICU patients are largely unknown.

**Methods:**

We performed a prospective questionnaire survey at two university hospital ICUs of the next-of-kin to 50 unconscious adult patients. They were asked to complete a questionnaire within 48 hours of the patients' acute ICU admission.

**Results:**

Forty-two relatives returned the questionnaire of which 41 were completed by direct family members and in one case by a friend to the patient.

The majority of relatives (36/42) were positive/positive with some scepticism towards performing drug trials in unconscious ICU patients and 30/42 would most likely accept trial-participation by their relative. The majority (30/42) agreed that they should decide if their relative was to participate in a drug trial and 24 of these found that the treating clinician/ICU consultant should also consent. The majority (27/42) found that deferred consent would be acceptable if there was a limited time frame for initiation of treatment, however 8 respondents found this unacceptable when the intervention was a new drug.

The majority of relatives stipulated that adherence to legislation, treatment benefit for the study patient and for future patients, no patient-risk or -discomfort and development of new drugs were important factors if their relative was to participate in an ICU drug trial. When questioned about doctors' motives for performing drug trials the wish for drug development and better patient care were highly rated among relatives.

**Conclusions:**

In general, relatives to unconscious ICU patients expressed positive attitudes to drug trials in the ICU and the inclusion of their relative in drug trials. Consent by next-of-kin and deferred consent was acceptable to the majority of relatives.

## Background

Researchers including critically ill patients into clinical trials are challenged by the ethical dilemma that many of these patients cannot give informed consent. In one study in the intensive care setting less than 3% of the included patients were able to consent prior to randomisation [1]. Therefore other procedures for consent are required when performing trials in this setting. In many countries patient relatives act as the legal representative to consent on behalf of the patient [2]. Consent rates by relatives when ICU patients are included into large interventional trials vary from 58 to 96% [3,4] and little is known of the attitudes to consent and perceived risk/benefit among relatives of critically ill patients. To investigate this, we performed a questionnaire survey among relatives of unconscious adult patients, who had been acutely admitted to the ICU.

## Methods

During two 4-week study periods, 50 relatives to acutely admitted adult ICU patients with impaired consciousness were asked to fill in a questionnaire within the first 48 hours of admission to the general ICUs at Rigshospitalet or Herlev Hospital in Copenhagen, Denmark. These hospitals receive patients from the whole of Denmark and suburban Copenhagen, respectively. The relative had to be the legal representative of the patient (i.e. nearest relative: spouse, parent, child or if the patient had no family, a friend) and were included by an ICU consultant (AP or JB), a research fellow (MI) or a research nurse, who gave information about the survey and the questionnaire to the relative. The relatives were encouraged to fill in the survey immediately and return the questionnaire to a member of the trial team or the patients contact nurse. There was no follow up of non-responders. Relatives who were unable to understand and read Danish or who had been asked for patient-inclusion into a research study were not invited to participate.

**Figure 1:**
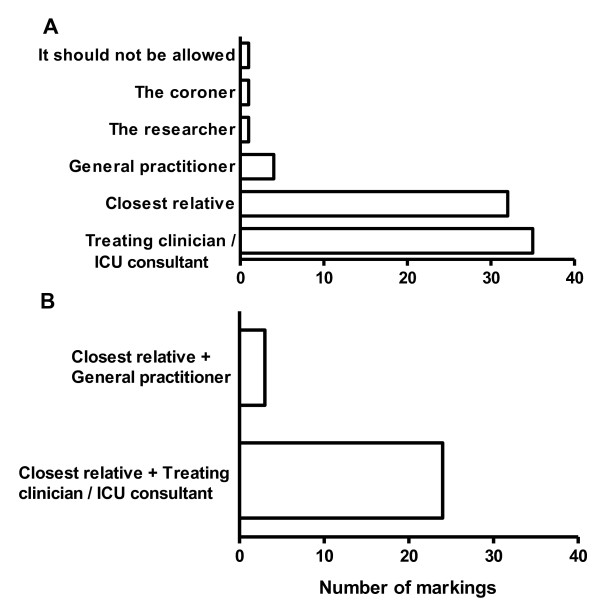
**Who are to decide if an unconscious patient is to participate in a drug trial? **Forty-two relatives of unconscious ICU patients were asked the above question. More than one answer could be marked. No relatives marked 'Do not know'. In **A**. all answer are displayed. In **B**. the answers of those relatives marking two answers (n = 27) are displayed.

The questionnaire was modified from a pre-existing questionnaire study of cancer patients and was adapted to be relevant to relatives to ICU patients [5]. Following the modifications to the questionnaire, it was preliminary trialled on five relatives to ICU patients, who were interviewed about its comprehensibility and user friendliness. This resulted in further adjustments and the final questions can be read in Results, Tables, Fig. 1 and Additional file [Supplementary-material S1] (translated from Danish).

We registered the sex, relation of the relative as well as patient age, diagnosis, reason for unconsciousness and sequential organ failure assessment (SOFA) score [6] at the time of questionnaire hand out. The survey fulfilled demands of Danish law, in which questionnaire-surveys are exempted from review by the ethical committee system.

Data were given as numbers of participants or medians with ranges where appropriate. No statistical analyses were performed and the sample size was chosen pragmatically.

## Results

Forty-two of the 50 relatives returned the questionnaire giving a response rate of 84%. Of these, 23 were spouses, 8 parents, 8 children, 2 were siblings and 1 was a friend of the ICU patient. The majority of relatives were females (27/42). The patients' diagnosis included severe sepsis (29), trauma (8), other acute surgical conditions (4) and hepatic failure (1) and all required mechanical ventilation. The median age of the patients was 55 (range 18 - 82) years and SOFA score at inclusion 9 (1 - 18). The reason for impaired conscious level was mainly sedation in 35 patients and encephalopathy in 7 patients.

### Relatives' knowledge about the purpose of drug trials

In response to the question 'What do you think is the purpose of performing drug trials?' the majority of respondents marked the answers 'to improve care of future patients' (38/42) and 'to increase doctors knowledge in general' (31/42), whereas 25/42 responded'to improve care of the patient in the trial'. Three respondents marked 'To reduce the total cost of treatment' and four marked 'So that drug companies can earn more money'.

### Relatives' attitudes to drug trials and consent

The majority of relatives were positive with some scepticism (36/42) to drug trials in unconscious ICU patients and would most likely accept (30/42) the participation of their relative in a trial. A minority (3/42) felt that drug trials should not be performed in unconscious patients (Table 1 &2).

**Table 1:** General attitudes to drug trials among 42 relatives of unconscious adult ICU patients

	Choices (percentages)			
Question	It is a natural part of any admission	Sceptical, but realize the necessity	Should not be allowed	Do not know
What do you think of drug trials in general?	15 (36)	25 (60)	1 (2)	1 (2)
What do you think of drug trials in unconscious patients?	9 (21)	27 (64)	3 (7)	3 (7)

**Table 2:** Attitudes to participation in drug trials among 42 relatives of unconscious adult ICU patients

	Choices (percentages)					
Question	Would accept	Would most likely accept	Hesitating	Would most likely not accept	Would not accept	Do not know
How is your attitude to your own potential participation in a drug trial?	11 (26)	23 (55)	5 (12)	2 (5)	1 (2)	-
How is your attitude if your relative, who is in intensive care, was to participate in a drug trial?	8 (19)	22 (52)	8 (19)	2 (5)	1 (2)	1 (2)

The majority(32/42) stated that they should decide if their relative was to participate in an ICU drug trial and 24 of these found that the treating clinician/ICU consultant should also give consent (Fig. 1). Very few wished that the researcher (1/42), general practitioner (4/42) or coroner (1/42) were to decide if their relative was to participate in a trial (Fig. 1).

Adherence to legislation, gain for the patient, gain for future patients, development of new drugs and no risk or discomfort for the patient were very important or important for the majority of respondents if their relative were to participate in a drug trial in the ICU (Table 3).

**Table 3:** Attitudes to drug trials among 42 relatives of unconscious adult ICU patients

Question	Grading (percentages)				
What would matter if your relative was to participate in a drug trial here in the intensive care unit?	Very important	Important	Minor importance	Not important	Do not know
'That current legislation was followed'	29	5	5	1	2
'That my relative was to gain directly from the trial'	22	13	6	0	1
'That future patients will gain from the trial'	23	15	1	0	3
'That new knowledge is accomplished to develop new therapies'	26	11	2	0	3
'That there is no risk by participation'	24	10	3	0	4
'That there is no discomfort by participation'	22	10	6	1	3
					
**What motives do you think doctors have when they perform drug trials?**					
'Wish to find new treatments'	39	1	0	0	2
'Wish to help patients'	38	3	0	0	1
'Wish to reduce overall cost of treatment'	0	10	15	12	5
'Wish to gain new knowledge'	33	7	1	0	1
'Wish to make carrier'	4	12	13	8	5
'Wish to earn money'	0	5	12	19	6

More than one answer could be marked.

Twenty-nine of the 42 respondents felt it acceptable to commence a drug trial before consent was obtained if the time window for initiation of treatment was narrow and therefore precluded obtaining consent. However 8 of the 29 would not endorse this if the trial was of a new drug and 2 would not for drug trials in general. Three relatives answered 'no' to this question on deferred consent and another 10 answered 'do not know'.

### Attitudes to doctors' motives for performing drug trials

Asked about doctors' motives for performing drug trials altruistic motives ('find new therapies', 'gain new knowledge' and 'help patients') were highly rated by the respondents (Table 3), whereas the majority thought that financial matters ('reduce costs of treatments' and 'earn money') and 'wish to make carrier' were of minor or no importance (Table 3).

## Discussion

The principle findings of this study were that the majority of relatives to unconscious ICU patients were positive or positive with some scepticism towards performing drug trials in unconscious ICU patients and towards the doctors/researchers performing such trials.

The majority would most likely accept participation in a drug trial by their relative, though on the premise that they should give consent if their relative was to participate. This is in line with results from a Swiss questionnaire survey where survivors of intensive care and their relatives wanted consent by the relative if the unconscious patient was to participate in a trial [7]. This notion is also supported by observations in a study of parents in a paediatric ICU, among whom the vast majority wanted to consent for their child's participation in a trial [8]. We know that relatives have difficulties when they are to express adult ICU patients' wishes for trial participation at least for hypothetical trials [9]. Furthermore up to 50% of relatives to ICU patients are hesitant in sharing the decision-making process for therapeutic interventions [10]. However, there appears to agreement between ICU patients and relatives on consent procedures in general as indicated by the aforementioned Swiss study [7].

In addition to adherence to legislation and no patient-risk or -discomfort, relatives had altruistic motives for patient participation in a drug trial. The relatives expected gain for the individual patient by trial participation, but from the answers we cannot know if they understood the concept of clinical trials. Therefore qualitative interviews of relatives would be of interest to assess if understanding for example the concept of randomisation would change their answers.

The majority of the relatives in our study expressed a wish for the treating clinician or ICU consultant to give consent to trial participation. It may be speculated that this is due to insecurity and a wish to share the responsibility with someone who knows the current medical status of the patient. In the Swiss study of ICU survivors and their relatives the majority wanted consent from two persons [7], but only 30% of the relatives wanted the intensivist to be one of these two. The reason for this discrepancy may be the timing of the two studies. In our study we included relatives within 48 hours of ICU admission whereas in the Swiss study the questionnaire was mailed to the relatives 3 years after ICU discharge.

The use of deferred consent in emergency situations may be supported by the data from the present study and those from recent studies of ICU patients and relatives [7,11]. An ethical analysis of emergency critical care research also supported the use of delayed consent [12]. On the other hand waiver of consent may not be supported, as it appeared to unacceptable to the relatives in the present study (not directly assessed) and a previous study [7]. Waiver of consent does increase inclusion rates [13], but high rates could likely be obtained using proxy consent by the treating clinician. Therefore, a consent procedure may be proposed for emergency situations where proxy consent is given by the treating clinician followed by deferred consent by the relative.

There are a number of strengths to this study. We prospectively included consecutive relatives within 48 hours of admission of their relative to the ICU. It is possible that attitudes of relatives change over time in the ICU, thus we have limited this potential confounder. Also, the relatively high response rate makes it likely that the overall results are representative for the whole cohort of relatives at least those of the including units, which covers city, suburban and countryside areas in Denmark.

There are also some weaknesses to the study. It was performed in a single Scandinavian country, so it is unknown if the results can be transferred to other countries or regions. This reduces the generalisability, because differences in culture and trust in the health care system and authorities in general are likely to influence the attitude to research. Due to limits in sample size, the influence of age and gender cannot be explored conclusively. As mentioned above, the attitudes presented here are those of the first 2 days after acute ICU admission. As we have no data on attitudes later in the admission period, the results are applicable only for those first days in the ICU. Also we have asked specifically about drug trials, so we do not know the attitude towards other types of studiesFinally, we did not include relatives who had been asked to consent for a patient trial. It may therefore be argued that the questions were theoretical for the relatives, therebyinfluencing their answers. On the other hand if the participants had been through the consent procedure for a trial prior to survey, the specific characteristics of this trial may also have influenced their answers. Taken together, a future survey should study a large sample in more countries and regions including both relatives that had and had not beenthrough a consent procedure prior to survey.

Hopefully, we will acquire more knowledge and data on ICU patients' and their relatives' attitudes towards trials, so that politicians can base future legislation on sound science. The present rigid consent procedures are likely to be a barrier for future research and scientific development in the critical care setting [14]. If we can match the process of consent to the attitudes and expectations of patients and relatives, more rational trial conduct is likely to result to the benefit of all involved in ICU trialsand society.

## Conclusions

In general, relatives to unconscious ICU patients expressed positive attitudes to drug trials in the ICU and the inclusion of their relative in drug trials. Consent by next-of-kin and deferred consent was acceptable to the majority of the relatives to unconscious ICU patients.

## Abbreviations

ICU: intensive care unit; SOFAscore: sequential organ failure assessment score.

## Competing interests

The authors declare that they have no competing interests.

## Authors' contributions

All authors were involved in study design, data collection and analysis and drafting and revision of the manuscript. All authors read and approved the final manuscript.

## Pre-publication history

The pre-publication history for this paper can be accessed here:

http://www.biomedcentral.com/1471-2253/10/6/prepub

## Supplementary Material

Additional file 1 **Questionnaire**. The questionaire used in the survey (translated form Danish) Click here for file
