# Supplementary material for: Attitudes to drug trials among relatives of unconscious intensive care patients
Source: BMC Anesthesiol. 2010 May 26;10:6. doi: 10.1186/1471-2253-10-6 (PMC2890661; doi:10.1186/1471-2253-10-6)
Supplement: Additional file 1 — Questionnaire. The questionaire used in the survey (translated form Danish) [file 1471-2253-10-6-S1.DOC]

Question 1. What do you think is the purpose of performing drug trials?

You may choose more than one answer

- To improve care of future patients
- To increase doctors knowledge in general
- To improve care of the patient in the trial
- To reduce the total cost of treatment
- So that drug companies can earn more money

Question 2. What do you think of drug trials in general?

- It is a natural part of any admission
- Sceptical, but realize the necessity
- Should not be allowed
- Do not know

Question 3. How is your attitude to your own potential participation in a drug trial?

- Would accept
- Would most likely accept
- Hesitating
- Would most likely not accept
- Would not accept
- Do not know

Question 4. What do you think of drug trials in unconscious patients? These patients are themself unable to accept or decline trial parcipation

- It is a natural part of any admission
- Sceptical, but realize the necessity
- Should not be allowed
- Do not know

Question 5. How is your attitude if your relative, who is in intensive care, was to participate in a drug trial?

- Would accept
- Would most likely accept
- Hesitating
- Would most likely not accept
- Would not accept
- Do not know

Question 6. Who are to decide if an unconscious patient is to participate in a drug trial? You may choose more than one answer.

Remember that all drug trials have to be approved by the Ethical Committee and the Drug Agency

- The closest relative
- The treating physician
- The ICU consultant
- The researcher
- The general practisioner
- The coroner
- It should not be allowed
- Do not know

Question 7. Should it be allowed to start a trial in unconscious patients if the condition makes no time to ask for permission? Permission will then have to be obtained later. This could be in cardiac arrest or stroke.

- Yes, unconditional
- Yes, but not for trials on new drugs
- Yes, but not for trial on drugs
- No
- Do not know

Question 8. What would be important if your relative was to participate in a drug trial here in the intensive care unit?

Mark the column you choose. You may choose more answers

|  | **Very important** | **Important** | **Minor importance** | Not important | Do not know |
| --- | --- | --- | --- | --- | --- |
| ‘That current legislation was followed’ |  |  |  |  |  |
| ‘That my relative was to gain directly from the trial’ |  |  |  |  |  |
| ‘That future patients will gain from the trial’ |  |  |  |  |  |
| ‘That new knowledge is accomplished to develop new therapies’ |  |  |  |  |  |
| ‘That there is no risk by participation’ |  |  |  |  |  |
| ‘That there is no discomfort by participation’ |  |  |  |  |  |

Question 9. What motives do you think doctors have when they perform drug trials?

Mark the column you choose. You may choose more answers

|  | **Very important** | **Important** | **Minor importance** | Not important | Do not know |
| --- | --- | --- | --- | --- | --- |
| ‘Wish to find new treatments’ |  |  |  |  |  |
| ‘Wish to help patients’ |  |  |  |  |  |
| ‘Wish to reduce overall cost of treatment’ |  |  |  |  |  |
| ‘Wish to gain new knowledge’ |  |  |  |  |  |
| ‘Wish to make carrier’ |  |  |  |  |  |
| ‘Wish to earn money’ |  |  |  |  |  |
